# Formation of neutrophil extracellular traps in mitochondrial DNA-deficient cells

**DOI:** 10.3164/jcbn.19-77

**Published:** 2019-11-12

**Authors:** Yutaka Takishita, Hiroyuki Yasuda, Mio Shimizu, Akane Matsuo, Akihiro Morita, Tomonari Tsutsumi, Masahiko Tsuchiya, Eisuke F. Sato

**Affiliations:** 1 Department of Biochemistry, Faculty of Pharmaceutical Sciences, Suzuka University of Medical Science, 3500-3 Minamitamagaki, Suzuka-city, Mie 513-8670, Japan; 2 Department of Anesthesiology, Osaka City University Medical School, 1-5-7 Asahi-machi, Abeno, Osaka 545-8586, Japan

**Keywords:** neutrophil extracellular trap, NETosis, mitochondria, peptidylarginine deiminase 4

## Abstract

Neutrophil extracellular trap (NET) formation plays an important role in inflammatory diseases. Although it is known that NET formation occurs via NADPH oxidase (NOX)-dependent and NOX-independent pathways, the detailed mechanism remains unknown. Therefore, in this study, we aimed to elucidate the role of mitochondria in NOX-dependent and NOX-independent NET formation. We generated mitochondrial DNA-deficient cells (ρ^0^ cells) by treating HL-60 cells with dideoxycytidine and differentiated them to neutrophil-like cells. These neutrophil-like ρ^0^ cells showed markedly reduced NOX-independent NET formation but not NOX-dependent NET formation. However, NET-associated intracellular histone citrullination was not inhibited in ρ^0^ cells. Furthermore, cells membrane disruption in NOX-dependent NET formation occurred in a Myeloperoxidase (MPO) and mixed lineage kinase domain like pseudokinase (MLKL)-dependent manner; however, cell membrane disruption in NOX-independent NET formation partially occurred in an MLKL-dependent manner. These results highlight the importance of mitochondria in NOX-independent NET formation.

## Introduction

Neutrophils are the first immune cells to respond to pathogen invasion and play a critical role in the subsequent immune response. They are the most abundant leukocytes in circulation and are first recruited to the infected sites. Here, the neutrophils are activated, and these activated neutrophils destroy the pathogens via generation of reactive oxygen species (ROS), phagocytosis, and formation of neutrophil extracellular traps (NETs).^(1)^ NETs are composed of DNA fibers, histones, and antimicrobial proteins such as myeloperoxidase (MPO) and neutrophil elastase released by neutrophils to capture and kill bacteria.^(2)^ NET formation has been reported in cancer, diabetes, and autoimmune diseases such as systemic lupus erythematosus and rheumatoid arthritis.^(3–4)^ Moreover, NETs bind to platelets, thereby causing vascular damage and arteriosclerosis.^(5)^ Although regulation of NET formation is considered to contribute to the prevention of exacerbation of the pathological condition, the detailed mechanisms underlying NET formation are not fully elucidated.

NET formation occurs NADPH oxidase (NOX)-dependent or NOX-independent mechanisms. NOX inhibitors were shown to inhibit both ROS generation and NET formation.^(6)^ Moreover, neutrophils isolated from patients with chronic granulomatous disease (CGD), which is characterized by impaired NOX activity, failed to generate ROS and did not show NET formation.^(7)^ Thus, the NOX-generated ROS play an important role in NET formation. However, it has been reported that neutrophils can extrude NETs via NOX-independent mechanisms in response to stimulants such as calcium ionophores (e.g., A23187).^(8,9)^ Furthermore, it was reported that the calcium ionophore-induced NOX-independent NET formation occurred via small conductance calcium-activated potassium channel protein 3 (SK3) and mitochondrial ROS.^(10)^

Mitochondria are multifunctional organelles that, produce ATP and regulate cell proliferation, differentiation, and oxidative signaling pathways. Therefore, mitochondrial dysfunction and oxidative stress in and around mitochondria have been implicated in pathogenetic mechanisms, including inflammation and autoimmune reactions. Using mitochondrial DNA (mtDNA)-deficient macrophages, we recently reported that mitochondria contribute to intracellular oxidative stress, which is responsible for stimulation of lipopolysaccharide-induced mitogen-activated protein kinase (MAPK) signaling to enhance cytokine release.^(11,12)^ However, the detailed relation between mitochondria and NOX-independent NET formation remains unclear. Therefore, in this study, we generated mtDNA-dificient (ρ^0^) cells created using dideoxycytidine (ddC) treatment in HL-60 cells and investigated the role of mitochondria in NOX-dependent and -independent NET formation.

## Materials and Methods

### Murine neutrophil isolation

Ten-week-old male C57BL/6 mice (SLC, Hamamatsu, Shizuoka, Japan) and gp91^*phox*^ knockout (KO) mice (Jackson Laboratories, Bar Harbor, ME) were used in the experiments. These mice were bred and housed individually in a specific pathogen-free barrier facility at 23°C with 12-h light/dark cycles. They were provided standard laboratory chow (CE-2, Oriental Yeast Co., Tokyo, Japan) and drinking water. This study was approved by the institutional animal ethics committee and was performed in strict accordance with the recommendations of the Guide for the Care and Use of Laboratory Animals of the Suzuka University of Medical Science (approval number: 21). For the isolation of infiltrating neutrophils, C57BL/6 and gp91^*phox*^ KO mice were intraperitoneally administered 2 ml of 2.98% thioglycollate (Becton, Dickinson and Company, Franklin Lakes, NJ) in PBS. At 4 h after the administration, neutrophils infiltrating the peritoneal cavity were collected using PBS. The isolated neutrophils were washed three times with PBS and then used for experiments.

### Cell culture and mtDNA-deficient cell (ρ^0^ cell) generation

The human promyelocytic leukemia cell line, HL-60 (RCB3683, RIKEN BioResource Center, Ibaraki, Japan) was cultured in RPMI 1640 medium (Nacalai, Kyoto, Japan) containing 10% (v/v) heat-inactivated fetal bovine serum and antibiotics in 5% CO_2_ humidified air at 37°C. ρ^0^ cells were produced by culturing HL-60 cells with 1 µM (final concentration) ddC for 7 days in the presence of uridine and pyruvic acid. HL-60 and ρ^0^ cells were differentiated into neutrophil-like cells by treatment with 1.25% dimethyl sulfoxide (DMSO) or 1 µM *all-trans* retinoic acid (ATRA) for 3 days, as described previously.^(13)^

### Quantification of extracellular DNA

Neutrophil-like HL-60 and ρ^0^ cells pretreated with or without 4-aminobenzoic acid hydrazide (ABAH; MPO inhibitor) for 3 h, MitoTEMPO (mitochondrial ROS scavenger) for 30 min, or necrosulfonamide [NSA; mixed lineage kinase domain like psedokinase (MLKL) inhibitor] for 30 min were seeded at 1 × 10^6^ cell/ml in 96-well plates. These cells were treated with 10 µM A23187 or 10 nM phorbol myristate acetate (PMA) for 3 h, whereas the murine neutrophils were treated with 10 µM A23187 or 1 µM PMA for 3 h. Then, all the cells were treated with 20 U/ml micrococcal nuclease (New England Biolabs Japan, Tokyo, Japan) for 20 min at 37°C. The DNA containing supernatants were collected after centrifugation at 200 × *g* for 8 min at 4°C. Extracellular DNA was transferred to a microwell plate, stained using SYTOX green, and quantified using SpectraMax^®^ (485 nm excitation; 525 nm emission; Molecular Devices Japan, Tokyo, Japan), and expressed as fold change with respect to the control.

### Quantification of NET-associated cell death (NETosis)

NETosis was quantified using a SYTOX green assay. Briefly, neutrophil-like HL-60 and ρ^0^ cells pre-treated with or without ABAH, MitoTEMPO, or NSA were seeded at 1 × 10^6^ cell/ml in 96 well plates and treated with 10 µM A23187 or 10 nM PMA. The murine neutrophils were treated with 10 µM A23187 or 25 nM PMA. The rate of NETosis was quantified hourly using SpectraMax^®^ (485 nm excitation, 525 nm emission) in the presence of SYTOX green. To calculate the relation of NETosis, fluorescence of the cells with 1% (v/v) Tritone X-100 was considered as 100% DNA, and NETosis at each time was showed at the % of total DNA.

### NET visualization

To observe NET formation, neutrophils and neutrophil-like HL-60 and ρ^0^ cells were seeded at 2 × 10^4^ cells in flexiPERM^®^ chamber inserts (OLYMPUS, Tokyo Japan) (pore size; 1.8 cm^2^) on a grass slide and incubated with 10 µM A23187 or 10 nM PMA. Then, the cells were incubated in SYTOX green for 5 min. Subsequent changes in fluorescence were observed using confocal microscopy.

### Western blotting

Cell samples were suspended in RIPA buffer and sonicated. Aliquots (15–30 µg) of the samples were loaded on SDS/PAGE gels. The electrophoresed samples were transferred on to PVDF or protein nitrocellulose membranes via a semi-dry transfer. The membranes were blocked by incubation in 5% non-fat milk in Tris-buffered saline with Tween 20. Then, the membranes were incubated with the following primary antibodies at 4°C overnight: anti-cytochrome c (1:1,000; 6H2.B4 556432; BD Bioscience), anti-mitochondria complex I–V (1:1,000; Total OXPHOS Rodent WB Antibody Cocktail ab110413; Abcam), anti-peptidylarginine deiminase type 4 (PAD4; 1:1,000; ab214810; Abcam), anti-TFAM (1:1,000; 18G102B2E11; Novus biologicals, Centennial, CO), anti-citrullinated histone H3 (1:1,000; ab5103; Abcam), and anti-MPO (1:1,000; ab9535; Abcam). The membranes were washed and incubated with secondary anti- mouse or rabbit IgG (1:2,000; Kirkegaard & Perry Laboratories, Inc., Gaithersburg, MD) at 25°C for 1 h. The protein bands were detected using ImmunoStar^®^ Zeta (Wako Pure Chemicals) and visualized using a LAS-4000 Mini imager (FUJIFILM, Tokyo, Japan).

### PCR and reverse transcription PCR (RT-PCR)

 Neutrophil-like HL-60 and ρ^0^ cells were harvested by centrifugation of 1 × 10^6^ cells per sample (and stored at –30°C). Nuclear DNA and mtDNA were isolated using the NucleoSpin^®^ Tissue kit (TaKaRa Bio, Shiga, Japan). Total cellular DNA concentrations were assayed using a Nano-Drop spectrophotometer (Thermo Fisher Scientific). For RT-PCR, total RNA was isolated from all the cell groups by using ISOGEN II (NIPPON GENE, Tokyo, Japan). The isolated RNA was then reverse transcribed using a ReverTra Ace^®^ kit (Toyobo, Osaka, Japan). The genomic DNA and 1st strand cDNA were subjected to PCR with the following primer sets: *Atp-6* (5'-atacacaacactaaaggacgaact-3', 5'-gaggcttactagaagtgaaaacg-3'), *p47*^*phox*^ (5'-agtagcctgtgacgtcgtct-3', 5'-acccagccagcactatgtgt-3'), *gp91*^*phox*^ (5'-tctcctcatcatggtgcaca-3', 5'-gctgttcaatgcttgtggct-3'), *p22*^*phox*^ (5'-gtttgttttgtgcctgctggagt-3', 5'-tgggcggctgcttgatggt-3'), *p67*^*phox*^ (5'-cgagggaaccagctgataga-3', 5'-catggaacactgagcttca-3'), *cytochrome c oxidase 1* (5'-tccttattcgagccgagctg-3', 5'-gggctgtga cgataacgttg-3'), *actin* (5'-agagctacgagctgcctgac-3', 5'-agcactgtgttg gcgtacag-3'), and *gapdh* (5'-gagtccttccacgataccaaag-3', 5'-cccctt cattgacctcaactac-3'). The PCR products were loaded on 1% agarose gels and stained with ethidium bromide (EtBr).

### Aminophenyl fluorescein (APF) assay

Neutrophil-like HL-60 and ρ^0^ cells were harvested by centrifugation of 1 × 10^6^ cells and washed with PBS. The harvested cells were treated with 10 µM APF just before 10 µM A23187 or 10 nM PMA stimulation. APF-stained cells were analyzed using flowcytometry (488 nm excitation; 575 nm emission; BD FACS Caliber; BD Biosciences).

### FACS analysis for CD11b

Cells (1 × 10^6^) were incubated at 0°C for 30 min with the anti-CD11b antibody (1:100; BD Biosciences), washed twice with PBS, and labeled with the FITC-conjugated goat anti-mouse IgG (BD Biosciences) at 0°C for 30 min. The cells were again washed with PBS and resuspended at 10^6^ cells/ml in 2% formaldehyde in PBS. FACS analysis was performed using BD FACS Caliber.

### Statistical analysis

Data are presented as mean ± SD from at least three experiments. Statistical analysis was performed using Student’s *t* test or one way ANOVA and post hoc Tukey test.

## Results

### NET formation in mouse neutrophils from gp91^*phox*^ KO mice

**Fig. 1:**
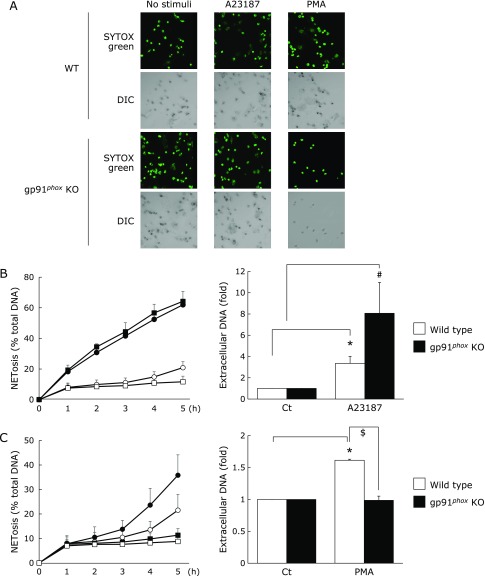
Neutrophil extracellular trap (NET) formation in neutrophils from gp91^*phox*^ knockout (KO) mice and wild-type (WT) mice. The gp91^*phox*^ KO and WT mouse neutrophil were stimulated with 10 µM A23187 (calcium ionophore) or 25 nM or 1 µM phorbol myristate acetate (PMA) for 3 h, and stained SYTOX green (5 µM), the cell-impermeable nucleic acid dye. (A) representative conforcal microscopy images showing NET formation; top panels: SYTOX green (DNA); bottom panels: differential interference contrast (DIC) images. (B) NETosis and extracellular DNA levels for A23187-stimulated neutrophils. (C) NETosis and extracellular DNA levels for PMA-stimulated neutrophil. (●): stimulated WT mice, (◯): unstimulated WT mice, (■): stimulated gp91^*phox*^ KO mice, (□): unstimulated gp91^*phox*^ KO mice. Date represent mean ± SD (*n* = 3). *p<0.01 A23187-treated cells (neutrophils from WT mice) vs Ct (unstimulated neutrophils from WT mice), ^#^*p*<0.05 A23187-treated cells (neutrophils from gp91^*phox*^ KO mice) vs Ct (unstimulated neutrophils from gp91^*phox*^ KO mice), ^$^*p*<0.01 PMA-treated cells (neutrophils from gp91^*phox*^ KO mice) vs PMA-treated cells (neutrophils from WT mice).

Using gp91^*phox*^ KO mice, we analyzed the NOX-dependent and NOX-independent NET formation induced by PMA and A23187, respectively. On PMA stimulation, the gp91^*phox*^ KO mouse neutrophils did not produce NOX-derived ROS, whereas the wild-type mouse neutrophils generated large amounts of ROS (data not shown). Compared to the wild-type mouse neutrophils, the gp91^*phox*^ KO mouse neutrophils did not release DNA into the extracellular space after PMA stimulation (Fig. 1A and C). In contrast, A23187 stimulation did not induce ROS generation in gp91^*phox*^ KO mouse neutrophils (date not shown); however, there was no difference between the extracellular DNA release in gp91^*phox*^ KO and control mouse neutrophils (Fig. 1A and B). These results indicate that A23187 induced NET formation in gp91^*phox*^ KO mice in a NOX-independent manner.

### Effect of MitoTEMPO on NETs formation of neutrophil-like HL-60 cells

**Fig. 2:**
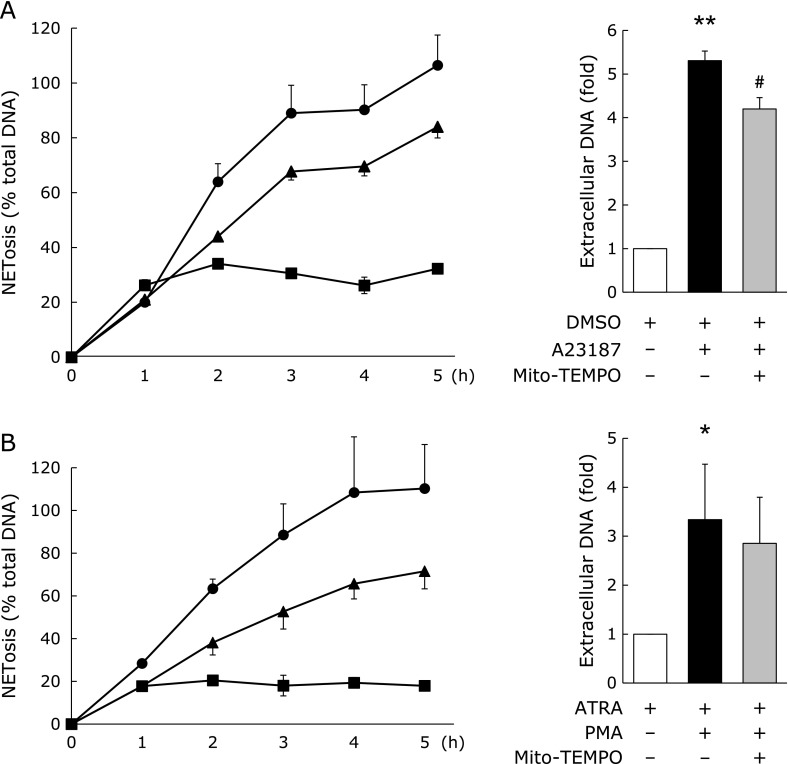
Effect of MitoTEMPO on NET formation in neutrophil-like HL-60 cells. (A) NETosis and extracellular DNA levels for A23187-treated cells. HL-60 cells were treated with 1.25% DMSO for 72 h for neutrophil differentiation. The resulting neutrophil-like cells were pretreated with 200 µM MitoTEMPO for 30 min and then treated with 10 µM A23187. (B) NETosis and extracellular DNA levels for phorbol myristate acetate (PMA)-treated cells. HL-60 cells were treated with 1 µM ATRA for 72 h for neutrophil differentiation. The resulting neutrophil-like cells were pretreated with 200 µM MitoTEMPO for 30 min and then treated with 25 nM PMA. (●): stimulated cells, (▲): stimulated and MitoTEMPO-treated cells, (■): unstimulated and untreated (control). *p<0.05, **p<0.01 vs control, ^#^*p*<0.01 vs A23187-stimulated cells.

ROS are produced not only by NOX, but also by mitochondria. Therefore, to evaluate the role of the mitochondrial ROS in the regulation of NET formation, we analyzed the effect of MitoTEMPO on NET formation in neutrophil-like HL-60 cells. MitoTEMPO is a mitochondria-targeted antioxidant that prevents mitochondrial oxidative damage. MitoTEMPO treatment slightly decreased the A23187- and PMA-induced NET formation (Fig. 2). Thus, MitoTEMPO did not completely suppress NET formation; therefore, we hypothesized that not only mitochondrial ROS but mitochondrial signaling is also involved in NET formation.

### Establishment of ρ^0^ cells from HL-60 cells

**Fig. 3:**
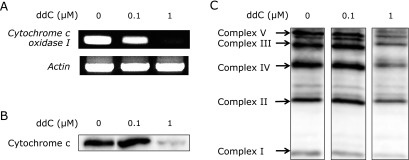
Establishment of mitochondrial DNA deficient cells (ρ^0^ cells) from HL-60 cells. HL-60 cells were treated with or without 0.1 or 1 µM ddC for 7 days. (A) Genomic PCR analysis showing the gene expression of *cytochrome c oxidase 1* and *actin*. (B) Western blot showing the protein expression of cytochrome c. (C) Western blots showing the protein expression of mitochondrial complexes.

Further, to explore the involvement of mitochondria in NOX-dependent and -independent NET formation (Fig. 2), we generated ρ^0^ cells from HL-60 cells. The previous method for generating ρ^0^ cells involved a long-term culture (1–2 months) of cells in the presence of EtBr (45 ng/ml).^(14)^ However, in this study, we used ddC, which prevents mtDNA replication, to establish ρ^0^ cells in a short period (1 week). Mitochondrial deficiency of the resultant cells was confirmed by analyzing the expression of cytochrome c oxidase, which is encoded by the mtDNA (Fig. 3). Genomic PCR analysis and western blotting revealed that HL-60 cells treated with 1 µM ddC for 7 days depleted gene and protein expression of cytochrome C (Fig. 3A and B). Moreover, the protein expression of mitochondrial complexes I–V was also markedly decreased in the ddC treated HL-60 cells (Fig. 1C). Thus, we established ρ^0^ cells from HL-60 cells using ddC for 1 week.

### Neutrophil-differentiation of ρ^0^ cells

**Fig. 4:**
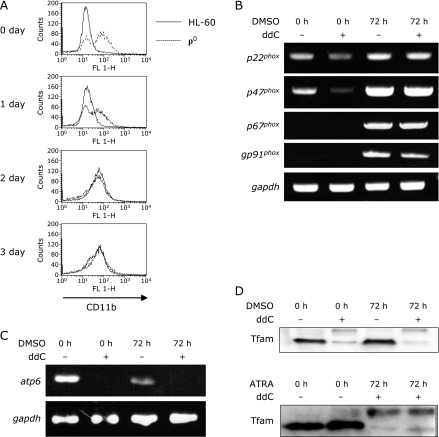
Characterization of neutrophil-like ρ^0^ cells. HL-60 and ρ^0^ cells were treated with 1.25% DMSO for 3 days. (A) Flow cytometry results showing CD11b expression from 0 day to 3 days after DMSO treatment. (B) RT-PCR results showing the expression of NADPH oxidase complex coponents (*p22*^*phox*^, *p47*^*phox*^, *p67*^*phox*^, *gp91*^*phox*^) before (0 day) and after (3 day) DMSO treatment. (C) Genomic PCR analysis showing *Atp6* expression before (0 day) and after (3 day) DMSO treatment. (D) Western blot showing TFAM expression before (0 day) and after (3 day) DMSO treatment.

To confirm the effect of mtDNA depletion on neutrophil differentiation, we treated HL-60 cells and ρ^0^ cells with 1.25% DMSO for neutrophil induction. We analyzed the expression of CD11b, a neutrophil surface antigen, using flow cytometry. CD11b was expressed on day 3 of differentiation in both HL-60 and ρ^0^ cells (Fig. 4A). There was no difference in the mRNA expression of NADPH oxidase complex components (gp91^*phox*^, p22^*phox*^, p47^*phox*^, and p67^*phox*^) of HL-60 and ρ^0^ cells on day 3 (Fig. 4B). *Atp6*, which is encoded in mtDNA, was markedly decreased in the DMSO-induced neutrophil-like ρ^0^ cells (Fig. 4C). The expression of TFAM, a transcription factor of mitochondria, also decreased in ρ^0^ cells and neutrophil-like ρ^0^ cells (Fig. 4D). these findings confirm that ρ^0^ cells remain mtDNA deficient even after differentiation and that there was no difference in neutrophil differentiation between HL-60 and ρ^0^ cells.

### NET formation in neutrophil-like HL-60 and ρ^0^ cells

**Fig. 5:**
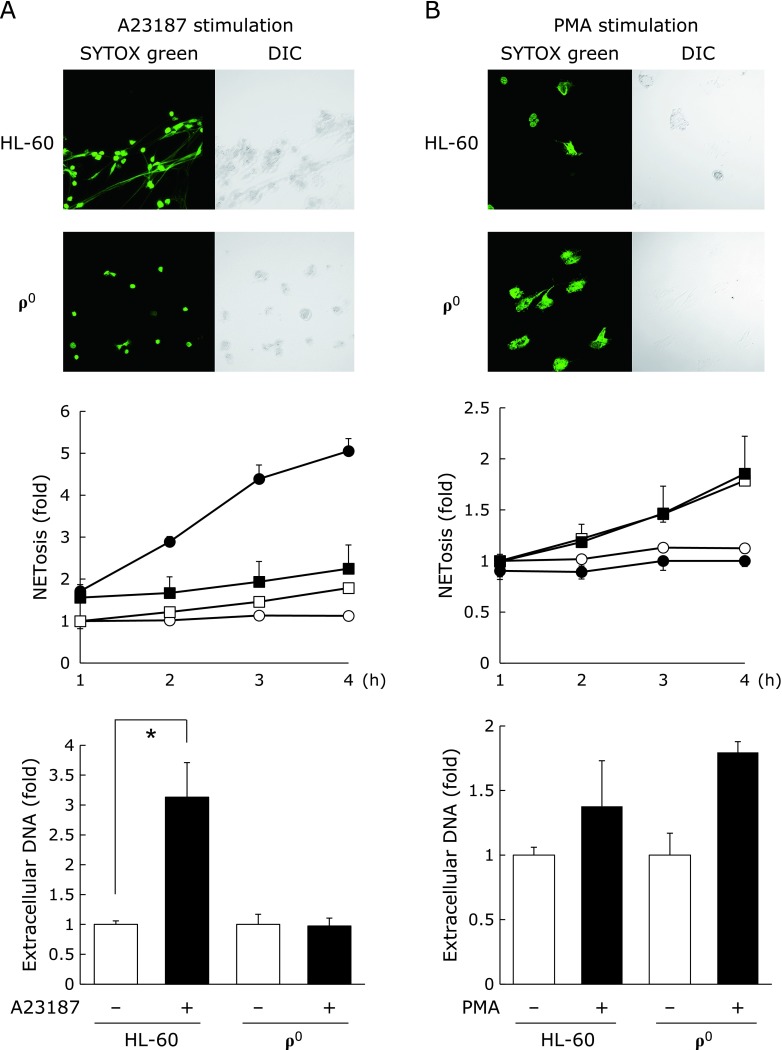
Analysis of NET formation of ρ^0^ cells in neutrophil-like HL-60 and ρ^0^cells. HL-60 and ρ^0^ cells were treated with 1.25% DMSO for 72 h, and then treated with (A) 10 µM A23187 or (B) 10 nM PMA for 4 h. Representative confocal microscopy images showing NETosis have been provided; top left panels:SYTOX green (DNA); top right panels: differential interference contrast (DIC) images. The middle and bottom panels show NETosis and extracellular DNA levels. (●): stimulated cells, (■): unstimulated cells, (□): stimulated ρ^0^ cells, (◯): unstimulated ρ^0^ cells. *p<0.05 vs control.

Next, to elucidate the role of mitochondria in NET formation and extracellular DNA release in neutrophil-like ρ^0^ cells. After A23187 stimulation, extracellular DNA release from neutrophil-like ρ^0^ cells was significantly lower than that from neutrophil-like HL-60 cells (Fig. 5A). However, there was no difference between the PMA-induced extracellular DNA release in neutrophil-like HL-60 cells and neutrophil-like ρ^0^ cells (Fig. 5B). These results suggest that mitochondrial function is essential for NOX-independent NET formation.

### Citrullination of histone H3 in neutrophil-like HL-60 and ρ^0^ cells

**Fig. 6:**
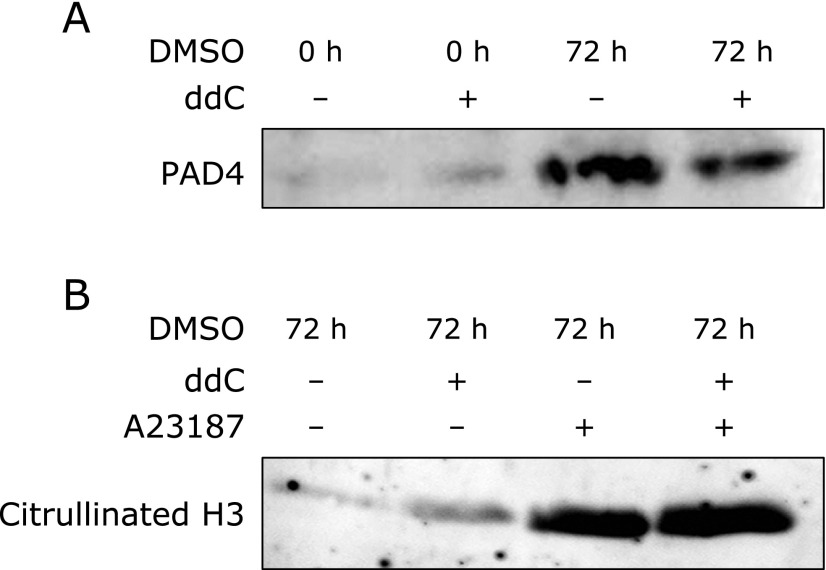
Peptidylarginine deiminase type 4 (PAD4) expression and histone H3 citrullination in neutrophil-like HL-60 and ρ^0^ cells. Neutrophil-like HL-60 and ρ^0^ cells were treated with 10 µM A23187 and nuclear extraction was performed. Western blotting was performed to analyze the expression of (A) PAD4 and (B) citrullination H3 (normalized to H3 protein levels).

Histone H3 citrullination plays a critical role in NET formation. Therefore, we analyzed H3 citrullination in neutrophil-like HL-60 and ρ^0^ cells using western blotting. Compared to the controls, A23187-stimulated neutrophil-like HL-60 and ρ^0^ cells showed increased expression of PAD4, an enzyme that converts histone arginine residues to citrulline (Fig. 6A), and increased expression of citrullinated H3 (Fig. 6B). Thus, although A23187 stimulation did not induce NET formation in neutrophil-like ρ^0^ cells, H3 citrullination was induced by A23187 stimulation. These results suggest that inhibition of NOX-independent NET formation did not affect PAD4 expression and histone citrullination.

### Cell membrane disruption during NET formation in neutrophil-like HL-60 cells

**Fig. 7:**
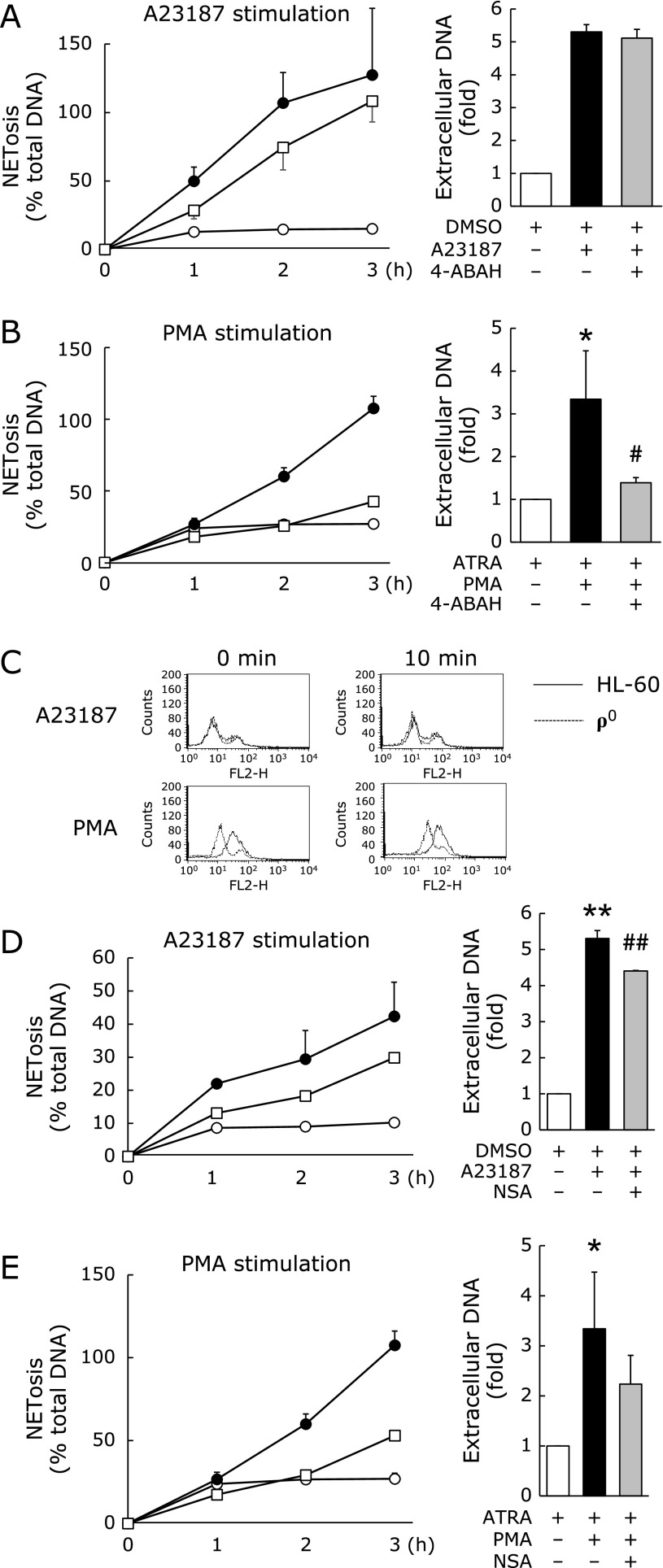
Analysis of plasma membrane disruption during NET formation in neutrophil-like HL-60 cells. (A) NETosis and extracellular DNA levels in A23187-stimulated HL-60 cells with or without ABAH treatment. HL-60 cells were treated with 1.25% DMSO for 72 h for neutrophil differentiation. The resulting neutrophil-like cells were treated with 500 µM ABAH for 3 h and then with 10 µM A23187. (B) NETosis and extracellular DNA levels for phorbol myristate acetate (PMA)-treated HL-60 cells with or without ABAH treatment. HL-60 cells were treated with 1 µM ATRA for 72 h for neutrophil differentiation. The resulting neutrophil-like cells were treated with 500 µM ABAH for 3 h and then with 25 nM PMA. (C) Aminophenyl Fluorescein (APF)-based flow cytometry analysis of HClO– generation before (0 min) and at 10 min after A23187 or PMA stimulation. (D) NETosis and extracellular DNA levels in A23187-stimulated HL-60 cells with or without necrosulfonamide (NSA) treatment. HL-60 cells were treated with 1.25% DMSO for 72 h for neutrophil differentiation. The resulting neutrophil-like cells were treated with 50 µM NSA for 30 min and then with 10 µM A23187. (E) NETosis and extracellular DNA levels for phorbol myristate acetate (PMA)-treated HL-60 cells with or without NSA treatment. HL-60 cells were treated with 1 µM ATRA for 72 h for neutrophil differentiation. The resulting neutrophil-like cells were treated with 50 µM NSA for 30 min and then with 25 nM PMA. (●): stimulated cells, (□): stimulated and inhibitor-treated cells, (◯): unstimulated and untreated (control). *p<0.05, **p<0.01 vs control, ^#^*p*<0.01, ^##^*p*<0.01 vs A23187-stimulated cells.

As H3 citrullination was not affected by mitochondrial deficiency, we next, investigated the cell membrane disruption mechanism in neutrophil-like HL-60 cells. HClO^−^ production by MPO was reported to be important for membrane disruption during NET formation.^(15,16)^ Therefore, we investigated the effect of ABAH, an MPO inhibitor, on NET formation. ABAH treatment did not suppress A23187-induced NET formation (Fig. 7A) but markedly suppressed PMA-induced NET formation (Fig. 7B). Therefore, we measured HClO^−^ generation using APF staining in neutrophil-like HL-60 and ρ^0^ cells. HClO^−^ generation was observed in both neutrophil-like HL60 and ρ^0^ cells after PMA stimulation but not after A23187 stimulation (Fig. 7C).

Therefore, to further investigate the other potential mechanism of membrane disruption in A23187- and PMA-induced NET formation, we investigated the effect of NSA, an MLKL inhibitor on NET formation in neutrophil-like HL-60 cells. NSA treatment suppressed both A23187- and PMA-induced NET formation (Fig. 7D and E). These data suggest the existence of different necroptosis mechanisms in NOX-dependent and NOX-independent NET formation.

## Discussion

In this study, we investigated NOX-dependent and -independent NET formation in mtDNA-deficient cells (ρ^0^ cells). We showed that mitochondria play an important role in the NOX-independent NET formation. Furthermore, membrane disruption in NOX-dependent NET formation occurred via MPO and MLKL, whereas that in NOX-independent NET formation was MPO-independent and was partially induced in an MLKL-dependent manner.

It is known that NET formation is induced in a NOX-dependent manner.^(1)^ Neutrophils from gp91^*phox*^ KO mice do not show NET formation in response to NOX-activating stimulants.^(17)^ In this study, the neutrophils from gp91^*phox*^ KO mice showed NET formation after A23187 stimulation but not after PMA stimulation. This indicates that NET formation occurs via NOX-independent mechanisms in gp91^*phox*^ KO mice. Moreover, inhibition of mitochondrial ROS production decreased NET formation induced via both NOX-dependent and -independent mechanisms (Fig. 2) suggesting that both the mechanisms involve mitochondrial ROS generation.

Previous studies demonstrated mtDNA deletion using EtBr.^(14)^ However, this method requires long-term culture (2 months) with EtBr, and the agent can potentially affect the genomic DNA of the cells. Therefore, in this study, we generated ρ^0^ cells from HL-60 cells using a novel method involving ddC.^(18)^ This innovative method required a short-term treatment (7 days) and provides a more efficient and highly reproducible alternative to generate ρ^0^ cells from HL-60 cells. Furthermore, the expression of CD11b, a differentiation marker, occurred earlier in ρ^0^ cells than in HL-60 cells (Fig. 4A), indicating that ρ^0^ cells might differentiate more rapidly than HL-60 cells.

NOX-independent NET formation after NADPH oxidase inhibition has been reported in human peripheral neutrophils.^(10)^ However, the mechanism of NOX-independent NET formation remains unclear. A recent study involving SK3 and mitochondrial ROS inhibitor suggested that calcium-activated NOX-independent NET formation is fast and mediated by SK3 and mitochondrial ROS.^(10)^ In this study, we investigated the role of mitochondrial pathway by using ρ^0^ cells, which lack the mitochondrial ROS generation and signal transduction.^(9,19)^ Our results were consistent with the previous results obtained using pharmacological approach, and confirm that mitochondrial signaling is essential for NOX-independent NET formation. Our findings also suggest that mitochondria do not affect NOX-dependent NET formation.

Several studies have reported the relation between MPO and NET formation.^(20–22)^ In particular, NET formation in MPO-deficient neutrophils was reported to be induced by calcium ionophore (ionomycin) but not by PMA.^(23)^ Furthermore, it was suggested that cell membrane destruction depends on MPO. Therefore, to clarify the involvement of MPO in cell membrane disruption mechanism, we analyzed the effects of pharmacological inhibition of MPO in this study. In neutrophil-like HL-60 cells, treatement with the MPO inhibitor ABAH significantly suppressed PMA-induced NET formation, but not A23187-induced NET formation (Fig. 7A and B). Interestingly, in both HL-60 and ρ^0^ cells, H3 citrullination occurred without membrane disruption (Fig. 6). Thus, membrane disruption occurred via different pathways during NOX-dependent and -independent NET formation. In NOX-dependent NET formation, cell membrane disruption occurred because of HClO^−^ generated by MPO.^(24)^ However, the involvement of MPO-generated HClO^−^ was not observed in NOX-independent NET formation; thus, the mechanism of cell membrane disruption in NOX-independent NET formation remained unclear. Recently, it was reported that anti-neutrophil cytoplasmic antibody (ANCA) induced NET formation via receptor-interacting protein kinase (RIPK) 1/3- and MLKL-dependent necroptosis.^(25,26)^ Another report also demonstrated that the RIPK-1 stabilizers necrostatin-1 or necrostatin-1s and the MLKL inhibitor NSA prevent monosodium urate crystal- or PMA-induced NET formation in human and mouse neutrophils.^(27)^ Therefore, we examined the effect of the NSA on NOX-dependent and -independent NET formation (Fig. 7). NSA partially inhibited both NOX-dependent and NOX-independent NET formation. Thus, membrane disruption in NOX-dependent NET formation involves both MPO-mediated HClO^−^ generation and MLKL activation, whereas membrane disruption in NOX-independent NET formation partially occurs via MLKL activity.

In conclusion, we generated mtDNA-deficient cells and showed that mitochondria, but not mitochondrial ROS, affected NOX-independent NET formation. Moreover, we show that cell membrane disruption in NOX-dependent NET formation occurs via both MPO- and MLKL-dependent mechanisms and that cell membrane disruption in NOX-independent NET formation partially occurs via an MLKL-dependent mechanism.

## Abbreviations

ABAH: 4-aminobenzoic acid hydrazide
APF: aminophenyl fluorescein
ATRA: *all-trans* retinoic acid
ddC: dideoxycytidine
DMSO: dimethyl sulfoxide
EtBr: ethidium bromide
KO: knockout
MAPK: mitogen-activated protein kinase
MLKL: mixed lineage kinase domain like pseudokinase
MPO: myeloperoxidase
mtDNA: mitochondrial DNA
NSA: necrosulfonamide
NET: neutrophil extracellular trap
NETosis: NET-associated cell death
NOX: NADPH oxidase
PAD4: peptidyl arginine deiminase 4
PMA: phorbol myristate acetate
ROS: reactive oxygen species
SK3: small conductance calcium-activated potassium channel protein 3
ρ^0^ cells: mitochondrial DNA-deficient cells
